# Advances in Vacuum Microwave Drying for Berries: Processing, Quality, and Future Perspectives for Sustainable Fruit Drying

**DOI:** 10.1111/1750-3841.71066

**Published:** 2026-04-14

**Authors:** Long Phuoc Lieu, Youngsoo Lee

**Affiliations:** ^1^ School of Food Science Washington State University Pullman Washington USA; ^2^ Department of Biological Systems Engineering Washington State University Pullman Washington USA

**Keywords:** anthocyanins, berries, drying kinetics, energy efficiency, modeling, phenolic compounds, quality preservation, sustainable food processing, vacuum microwave drying

## Abstract

Berries are valued for their vibrant color, delicate texture, and high levels of bioactive compounds; however, their soft tissues and high moisture content make them highly perishable, resulting in rapid postharvest deterioration and limited storage stability. Drying is a practical approach to extend their shelf life, although many conventional drying techniques expose berries to prolonged heating and oxygen, which leads to flavor loss, pigment degradation, and structural collapse. Vacuum microwave drying (VMD) is considered an advanced drying technology that unites rapid volumetric microwave heating with low‐pressure dehydration to achieve high‐quality dried fruits in a fraction of the time required by conventional drying methods. This review provides a comprehensive analysis of recent progress in the application of VMD to berry processing, focusing on the effects of microwave power, vacuum pressure, product temperature, and pretreatments on drying kinetics, nutrient retention, and microstructural quality. Comparative studies demonstrated that VMD produces berries with superior color, texture, and rehydration ability, comparable to or even better than those obtained by freeze drying, while consuming substantially less energy. Despite its potential, industrial adoption remains limited by challenges in energy distribution, microwave penetration depth, and process scalability. Recent studies increasingly emphasize the potential of hybrid drying strategies and enhanced process control as promising directions to improve the reliability and efficiency of VMD applications. Therefore, VMD represents a promising technology that bridges scientific innovation and industrial practice, offering an efficient and low‐energy pathway toward the production of high‐value dried fruits with improved nutritional quality and sensory attributes.

**Abbreviations**
ANN: artificial neural network
CD: convective drying
FD: freeze drying
HACD: hot‐air convective drying
OD: osmotic dehydration
PEF: pulsed electric field
RSM: response surface methodology
SEC: specific energy consumption
US: ultrasound
VMD: vacuum microwave drying

## 1 Introduction

Berries such as strawberries, blueberries, raspberries, and cranberries occupy a unique position in the global fruit market due to their intense color, delicate flavor, and remarkable nutritional profile (Vahapoglu et al. 2021). Berries are widely recognized as significant sources of bioactive compounds such as anthocyanins, flavonoids, and vitamin C, which not only define their sensory characteristics but also contribute to significant health‐promoting properties as functional foods (Bezerra et al. 2024). Despite their value, berries are among the most perishable fruits. Their high moisture content, tender epidermal tissues, and active enzymatic systems lead to rapid deterioration, causing substantial postharvest losses and constraining their distribution to distant markets (Iñiguez‐Moreno et al. 2023; Oliveira et al. 2019; Pott et al. 2020). Addressing this challenge requires advanced preservation strategies capable of extending shelf life without compromising the nutritional and sensory qualities that make berries so desirable. Among available preservation approaches, drying remains one of the most practical and widely applied strategies for stabilizing berries and improving their marketability beyond the harvest season.

Drying is one of the oldest and most effective preservation techniques; however, the methods traditionally applied to berries are fraught with limitations (Calín‐Sánchez et al. 2020). Hot‐air drying and vacuum oven drying are widely used but often involve long processing times and high thermal loads, which result in significant degradation of heat‐sensitive phytochemicals, undesirable changes in color and texture, and a marked reduction in rehydration capacity (Akcicek et al. 2023; ElGamal et al. 2023; Radojčin et al. 2021). Freeze drying (FD) has been regarded as the benchmark for high‐quality dried products since it can preserve cellular structure and minimize nutritional losses, but its high energy requirements, high capital cost, and lengthy processing cycles make it challenging for large‐scale applications (Al Faruq et al. 2025; Nwankwo et al. 2023; Silva‐Espinoza et al. 2019). The limitations of these approaches underscore the need for innovative drying technologies that can bridge the gap between product quality retention and process efficiency. Consequently, increasing research attention has been directed toward emerging drying technologies, including infrared drying, ultrasound (US)‐assisted drying, and various hybrid drying approaches such as hot air–infrared, infrared–vacuum, microwave–infrared, and microwave–vacuum systems (D. Huang et al. 2021).

**FIGURE 1:**
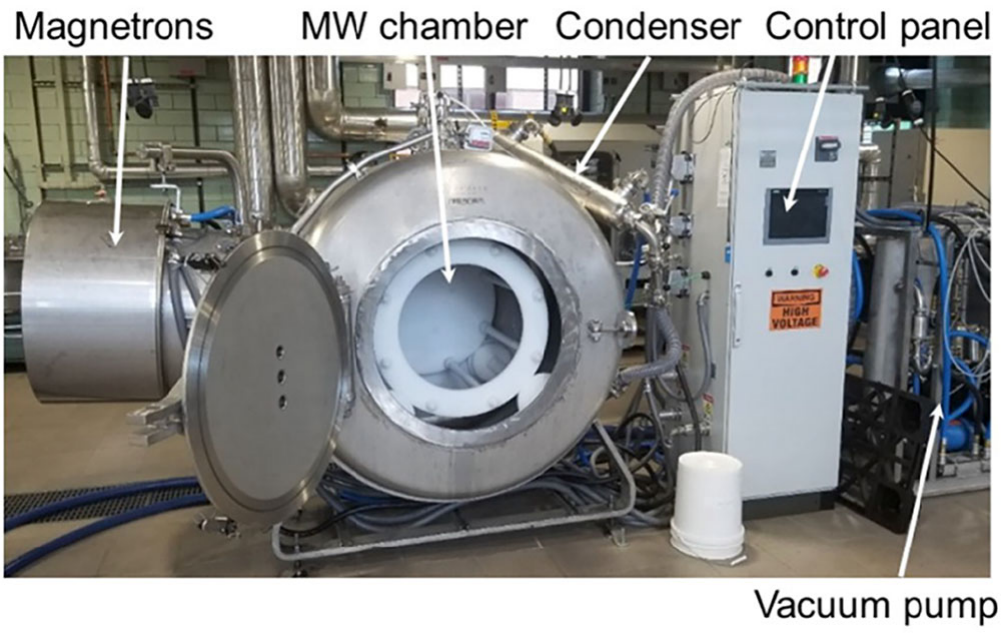
A pilot‐scale vacuum microwave drying (VMD) system, showing the microwave chamber, magnetrons, condenser, vacuum pump, and control panel. Reproduced from Gong et al. (2025) under the Creative Commons Attribution (CC BY 4.0) license.

Vacuum microwave drying (VMD; Figure 1) is considered a promising drying technique that addresses many of these concerns. By combining the advantages of reduced pressure with the rapid volumetric heating by microwaves, this technique accelerates moisture removal through internal vapor generation at lower boiling points, reducing drying times drastically while mitigating thermal damage. The rapid evaporation of water under vacuum conditions often creates porous structures that enhance crispness and improve rehydration behavior, while the minimal oxygen exposure and moderate temperatures help preserve anthocyanins, phenolics, volatile compounds, and vitamins that are otherwise lost during conventional drying processes (Gul et al. 2024; Wojdyło et al. 2014; Xing et al. 2025; L. Zhang et al. 2023). Comparative studies increasingly demonstrate that VMD can achieve quality parameters comparable to, and in some cases superior to, FD, while offering higher operational speed, improved energy efficiency, and lower operational cost (Calín‐Sánchez et al. 2020; Taghinezhad et al. 2023).

Despite these advantages, the broader adoption of VMD for berries faces several key challenges. Issues such as nonuniform microwave energy distribution, limited penetration depth, and process scalability remain as barriers to industrial implementation (Joardder and Karim 2025; Z. Y. Li et al. 2011; Wardhani et al. 2022). Furthermore, the structural heterogeneity of different berry species requires product‐specific optimization of pretreatments, drying parameters, and process control strategies. Recent studies have begun to explore solutions, including pulsed microwave energy, staged pressure profiles, hybrid methods that combine vacuum microwave with hot‐air or infrared treatments, and advanced monitoring systems that allow real‐time control of moisture removal and energy input (Bagchi Banerjee and Janghu 2023; Pu and Sun 2017; Zang et al. 2023). These innovations point to a clear trajectory toward more reliable, efficient, and scalable applications of VMD technology in berry processing. However, despite the growing number of experimental studies, the available literature remains fragmented, and a comprehensive synthesis of the mechanisms, operational parameters, drying kinetics, and quality outcomes of VMD, specifically for berry matrices, is still lacking.

This review, therefore, aimed to provide a comprehensive analysis of the principles, mechanisms, and applications of VMD for berries. This review includes examination of the underlying heat and mass transfer phenomena, evaluation of the effects of processing parameters on product quality, and the retention of bioactive compounds, as well as comparisons with other conventional and advanced drying techniques. It further highlights progress in system design, modeling, and process optimization, while also identifying knowledge gaps and practical barriers that must be overcome for industrial applications. By consolidating current knowledge and mapping future research directions, this review aims to establish a clear framework for understanding and advancing VMD as a sustainable and high‐performance technology for premium berry products.

## 2 Mechanism of VMD

**FIGURE 2:**
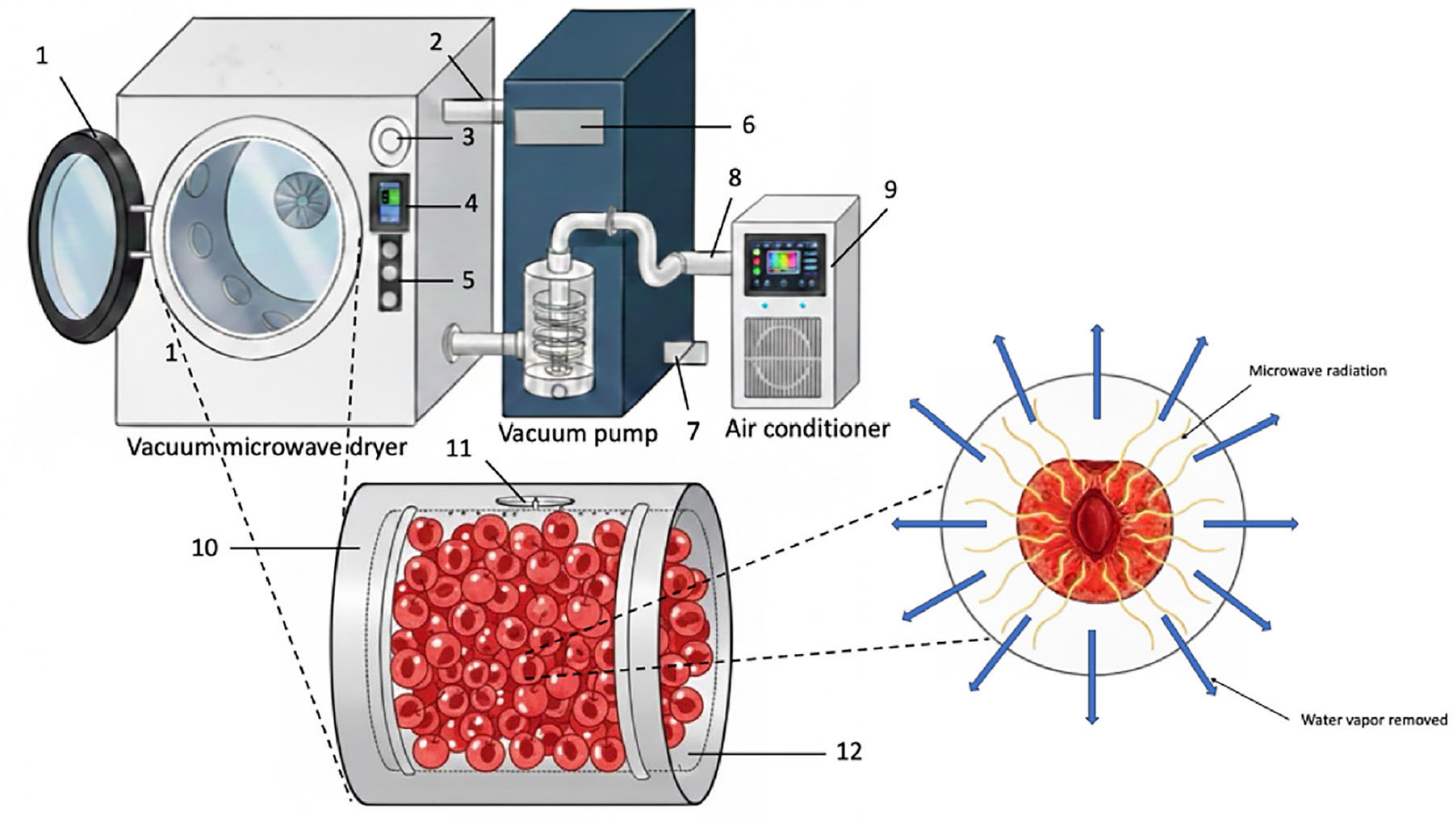
Schematic illustration of a vacuum microwave drying (VMD) system and its working mechanism for sweet cherries. Components: (1) operation door; (2) stainless‐steel pipe connecting the microwave vacuum chamber to the vacuum pump; (3) pressure detector; (4) system controller (digital microwave power controller) and temperature detector; (5) start/stop and door‐opening switches; (6) vacuum pump control box; (7) stainless‐steel drain pipe for water removal from the vacuum pump; (8) stainless‐steel pipe connecting the vacuum pump to the air conditioner; (9) air conditioner control box; (10) drying chamber containing sweet cherries; (11) magnetrons; and (12) 360° rotating load basket. Modified from Hirun and coworkers (2014).

Figure 2 illustrates that VMD relies on a dual mechanism that integrates reduced‐pressure evaporation with rapid volumetric microwave heating. Under vacuum, the boiling temperature of water significantly decreases, which enables internal vaporization at relatively low product temperatures, typically below 55°C in the final drying stage. Microwaves penetrate the material and selectively interact with polar water molecules through dielectric polarization (Chandrasekaran et al. 2013; Datta 2001; M. Zhang et al. 2006). The continuous oscillation of these molecules produces heat throughout the entire volume rather than only at the surface, establishing a strong internal driving force for moisture removal (Charoenrein and Preechathammawong 2010). As vapor pressure inside berry tissues rapidly increases, moisture is expelled outward through microcapillaries, enabling a high mass transfer rate that is fundamentally different from conventional surface‐limited drying (Drouzas et al. 1999; Suo et al. 2025). Lower oxygen levels in the vacuum chamber suppress oxidative reactions responsible for pigment bleaching, vitamin degradation, and flavor loss. This protection against oxidative reactions is beneficial for berries that contain anthocyanins, phenolics, and aromatic compounds that are highly sensitive to heat and oxygen exposure (Giri and Prasad 2007; Lachowicz et al. 2019; Yongsawatdigul and Gunasekaran 1996). At the same time, the swift evaporation of entrapped moisture can expand or preserve open cellular structures, leading to improved porosity and superior rehydration capacity of dried berries. By minimizing thermal collapse, VMD supports retention of the natural appearance and desirable texture (Guo et al. 2025; L. Huang et al. 2012).

The drying process in VMD is dynamic and moisture dependent. High water content at the initial stage promotes strong microwave absorption, resulting in intense internal heating and fast drying. As moisture decreases, the dielectric properties of a product change, and microwave penetration becomes less uniform. Without adequate control, localized overheating or temperature spikes may occur, causing hardened surfaces, internal cracks, or degradation of thermolabile nutrients (Z. Y. Li et al. 2011; Vadivambal and Jayas 2010). Therefore, the success of VMD heavily depends on precise control of microwave power, real‐time temperature, and staged pressure application that balance drying acceleration with structural protection.

From an energy requirement standpoint, VMD is fundamentally more efficient than convective drying (CD) because the applied energy is delivered directly to the water molecules instead of heating the surrounding air or equipment (Liang et al. 2024). However, limited microwave penetration depth and nonuniform electromagnetic field distribution remain critical engineering barriers, especially in whole berries with spherical geometry and waxy cuticles that impede energy transmission (Joardder and Karim 2025). Recent advances in cavity design, rotating bed systems, adaptive power modulation, and multisensor monitoring help overcome these constraints, enabling more homogeneous heating patterns and consistent product quality (Dehghannya and Habibi‐Ghods 2025; González‐Cavieres et al. 2021).

Therefore, the mechanism of VMD centers on the interplay of rapid internal heating, reduced‐pressure evaporation, and microstructural stabilization, providing a unique pathway to produce high‐quality dried berries with reduced processing time and minimum quality degradation. Continued progress in equipment optimization and process modeling is essential to fully unlock the VMD's potential industrial applications.

## 3 VMD on Berries

VMD has gained significant attention as an advanced dehydration technique for thermally sensitive fruits such as berries. Unlike conventional drying methods such as hot‐air drying or FD, VMD provides a unique combination of speed, energy efficiency, and product quality. Under vacuum, the boiling point of water decreases significantly, allowing dehydration to occur at temperatures typically below 55°C, thereby protecting thermolabile phytochemicals such as anthocyanins, phenolic contents, and vitamins (Wojdyło et al. 2014; Yongsawatdigul and Gunasekaran 1996; Zielinska and Michalska 2016). Moreover, the volumetric nature of microwave heating ensures uniform temperature distribution throughout the fruit matrix, preventing case hardening and structural collapse that are common in CD (Nowacka et al. 2019; Zhou et al. 2021). In recent years, VMD has been successfully applied to a wide range of berry species, including strawberries (*Fragaria ananassa*), blueberries (*Vaccinium corymbosum*), cranberries (*Vaccinium macrocarpon*), and sour cherries (*Prunus cerasus*). The technology is also adaptable to hybrid configurations such as osmotic dehydration followed by VMD (OD + VMD), convective pre‐drying combined with VMD (CD + VMD), or hot‐air convective drying followed by microwave–vacuum finishing (HACD + VMD). These multistage systems integrate the advantages of each method, leading to shorter drying times, improved rehydration capacity, and superior retention of phenolics and anthocyanins compared with single‐stage drying (Z.‐L. Liu et al. 2020; Ropelewska et al. 2023). This comparative overview provides a foundation for the detailed discussion in the subsequent sections on the drying parameters, drying kinetics, and quality attributes of VMD‐processed berries.

### 3.1 Operational Parameters of VMD

The performance of VMD systems and the quality of dried berries are highly dependent on operational parameters, including microwave power, vacuum pressure, temperature, pretreatments, and product geometry. Optimization of these factors is crucial for balancing rapid dehydration time, nutrient retention, and product texture. A summary of representative studies on VMD of berries is presented in Table 1, which outlines the diversity of materials, process conditions, and outcomes reported in the literature.

**TABLE 1:** The effects of operational parameters of vacuum microwave drying (VMD) on berries.

Reference	Type/process	Material	Sample geometry	Pressure (kPa)	Power strategy	Key results
Motavali et al. 2013	VMD	Sour cherry	Whole (pitted)	20–80	360–1200 W	Midilli model best fit; lowest SEC (12.9 kWh/kg) at 1200 W and 20 kPa; drying 15–79 min
Wojdyło et al. 2014	VMD	Sour cherry (cv. Turgieniewka)	Whole (pitted)	4–6	Reduced 480 to 120 W	Drying 23–48 min; phenolics 7587 mg/kg dm; anthocyanin retention 70%–90%; color and antioxidant activity comparable to freeze drying
Nowicka et al. 2015	OD + CD + VMD	Sour cherry (pitted, OD‐treated)	Whole (pitted)	4–6	360 W (finish)	OD 120 min + CD 90 min + VMD preserved bioactives and reduced total drying time by 50%
Zielinska and Michalska 2016	HACD + VMD	Blueberry (*Vaccinium corymbosum*)	Whole	5 ± 1	1.3 W/g	Drying reduced by 55%; effective diffusivity 3.11 × 10^−^^9^ m^2^/s; anthocyanin retention 30%; optimal antioxidant capacity at HACD 90°C + VMD
Zielinska et al. 2018	OD + VMD with microwave pretreatment	Cranberry (*Vaccinium macrocarpon*)	Whole	5 ± 1	100–800 W pretreatment	Microwave–vacuum pretreatment accelerated mass transfer during osmotic dehydration; improved retention of phenolics, flavonoids, and antioxidant activity compared with conventional drying
Zielinska and Zielinska 2019	VMD	Cranberry (*Vaccinium macrocarpon*)	Whole	5 ± 1	100–500 W (stepwise)	Drying time reduced from 91 to 10 min; best retention of total phenolics, flavonoids, and anthocyanins at 300 W; antioxidant activity comparable to freeze drying
Nowacka et al. 2019	VMD with US, PEF, and OD pretreatments	Cranberry (*Vaccinium oxycoccos*)	Whole	6.5	150 W	Drying 25–38 min vs. 13 h CD; *D*_eff_ = 2.0–2.8 × 10^−^^9^ m^2^/s; ultrasound and PEF increased phenolic and vitamin C retention
de Bruijn et al. 2016	VMD	Strawberry (*Fragaria ananassa*)	Whole	6	700 W	Moisture reduced from 90.4% to 29.3%; Δ*E* ≈ 5; polyphenols/flavonoids ∼53% retained; shelf life ≈ 68 days
Bórquez et al. 2015	VMD with temperature control	Strawberry (*Fragaria ananassa*)	Whole; halved and diced (comparative)	6–21	700→119 W (auto)	Δ*E* = 5.8; SEC 1.7–2.3 Wh/g; efficiency 50%–54%; quality close to freeze‐dried; 13× higher efficiency vs. manual mode
Z.‐L. Liu et al. 2020	HACD + VMD (with pretreatments)	Cranberry	Whole	5 ± 1	150 W	Drying 243–374 min HACD + VMD; best with sonication/freezing pretreatments; high polyphenols (27.5 mg GAE/g DM) and antioxidant capacity (33–47 mg TE/g DM).
Ropelewska et al. 2023	Simultaneous OD + VMD	Sour cherry (three cultivars)	Whole (pitted)	3	Stepwise 100–300–250 W	Drying ≈ 1 h; *a*_w_ ≈ 0.4; sensory 8.8–8.9/10; vivid color and texture preserved
Cong et al. 2023	VMD	Mulberry (*Morus alba*)	Whole	20–80 kPa vacuum level equivalent	1.82–5.45 W/g	Optimal drying obtained at ∼50°C surface temperature and 5.45 W/g; effective diffusivity ranged 4.98 × 10^−8^ to 3.81 × 10^−7^ m^2^/s; loose porous structure formed during drying

Abbreviations: *a*_w_, water activity; CD, convective drying; *D*_eff_, effective moisture diffusivity; HACD, hot‐air convective drying; OD, osmotic dehydration; PEF, pulsed electric field; SEC, specific energy consumption; US, ultrasound; VMD, vacuum microwave drying.

#### 3.1.1 Microwave Power

Microwave power is the dominant factor controlling drying kinetics and heat distribution. Increasing the power input raises the internal temperature and accelerates water evaporation but may also risk overheating or pigment loss if not carefully managed. Motavali et al. (2013) demonstrated in sour cherries that increasing power from 360 to 1200 W under 200–800 mbar shortened drying time from 79 to 15 min, while the Midilli model best described the drying behavior. The specific energy consumption (SEC) reached its lowest value at 12.9 kWh/kg when the process was conducted at 1200 W and 200 mbar (Motavali et al. 2013). Similarly, Wojdyło et al. (2014) reported that a gradual reduction in power from 480 to 120 W at 4–6 kPa allowed the sour cherries to retain 70%–90% of their anthocyanins and more than 7500 mg/kg of phenolics, maintaining vivid color and high antioxidant activity (Wojdyło et al. 2014). A similar pattern was observed in cranberries. Zielinska and Zielinska (2019) found that moderate microwave power around 300 W at 5 kPa provided the best balance between drying rate and preservation of total phenolics, flavonoids, and anthocyanins, whereas power above 450 W promoted excessive temperature rise and color deterioration (Zielinska and Zielinska 2019). Similarly, Zielinska et al. (2018) also showed that microwave–vacuum pretreatment of cranberries prior to OD significantly influenced mass transfer and final product quality, with lower power levels favoring the preservation of phenolics, anthocyanins, and antioxidant activity (Zielinska et al. 2018). Comparable observations were reported by Cong et al. (2023) for mulberries, where microwave–vacuum drying parameters strongly affected product temperature, water diffusion, and textural properties (Cong et al. 2023). These findings indicate that although higher microwave power accelerates moisture removal and shortens drying time, excessive power may lead to thermal degradation of bioactive compounds. Therefore, optimized power control is essential to balance drying efficiency with the preservation of anthocyanins, phenolics, and color stability during VMD processing of berries.

#### 3.1.2 Vacuum Pressure

Vacuum level plays a decisive role in determining both the boiling point of water and the overall mass transfer dynamics during VMD. Lowering the pressure decreases the boiling point, which accelerates moisture removal via phase change and simultaneously reduces oxidative degradation of sensitive compounds. However, when the vacuum pressure becomes excessively low, rapid vapor expansion within the cellular matrix can lead to structural collapse and localized overheating (Hu et al. 2025). In berries, which possess thin skins, high intracellular moisture, and fragile parenchymal tissue, excessively low vacuum levels can promote cell wall rupture, berry collapse, and surface scorching, ultimately impairing texture, color uniformity, and retention of anthocyanins (Wojdyło et al. 2014; M. Zhang et al. 2006).

Most studies have reported that maintaining the pressure within the range of 4–6 kPa yields the most favorable balance between drying efficiency and product quality (Wojdyło et al. 2014; Zielinska et al. 2017). Under these conditions, the diffusion of moisture occurs uniformly, preventing structural collapse and ensuring even heating. In the case of blueberries, Zielinska et al. (2016) observed that a pressure of approximately 5 ± 1 kPa produced the highest effective moisture diffusivity of 3.11 × 10^−9^ m^2^/s and the best retention of phenolic compounds (Zielinska and Michalska 2016). Conversely, Motavali et al. (2013) demonstrated that increasing the vacuum range from 200 to 800 mbar (equivalent to 20–80 kPa) significantly prolonged drying time and raised energy consumption. These observations confirm that maintaining a strong vacuum below 10 kPa is crucial for achieving rapid and energy‐efficient dehydration of berries (Motavali et al. 2013).

#### 3.1.3 Temperature and Process Time

Temperature during VMD is not controlled directly but emerges as a function of the selected microwave power and vacuum pressure. In most cases, product temperatures between 45°C and 65°C are sufficient to achieve effective dehydration while preventing thermal degradation of heat‐sensitive compounds such as anthocyanins and vitamin C. De Bruijn et al. (2016) found that strawberries dried at 700 W under 6 kPa lost about 90% of their initial moisture within 3 h while retaining color close to the fresh state (Δ*E* ≈ 5) and more than half of the original polyphenols (de Bruijn et al. 2016). In blueberries, Zielinska and Michalska (2016) showed that hybrid HACD + VMD reduced total drying time by about 55% relative to hot‐air drying alone, while improving antioxidant capacity and maintaining acceptable anthocyanin retention (Zielinska and Michalska 2016). Likewise, Zhou et al. (2021) demonstrated that combining HACD at 80°C with a short microwave–vacuum stage at 150 W and 5 kPa reduced total drying time by more than half. Despite the shorter duration, the hybrid process maintained polyphenol content up to 27 mg GAE/g dry matter and preserved antioxidant activity (Zhou et al. 2021). These studies suggest that moderate‐temperature, short‐duration microwave stages are sufficient for effective drying while maintaining the nutritional integrity of berries.

#### 3.1.4 Pretreatments and Product Geometry

Pretreatment methods are frequently employed to enhance mass transfer and improve color stability during VMD by altering the cell wall structure and osmotic potential of the fruit tissue. Nowicka et al. (2015) demonstrated that OD of sour cherries in 40 °Brix apple concentrate at 40°C for 120 min, followed by CD at 50°C (air velocity 0.8 m/s) for 90 min and a short VMD stage (360 W, 4–6 kPa), effectively shortened total drying time and preserved high phenolic content. The improved retention of phenolic compounds was attributed to substantial moisture reduction during OD (>50%), which enabled shorter exposure to hot‐air drying, combined with rapid moisture removal under vacuum–microwave conditions that limited oxygen availability and thermal stress during the final drying stage (Nowicka et al. 2015). Similarly, Nowacka et al. (2019) and Z.‐L. Liu et al. (2020) found that US and pulsed electric field (PEF) pretreatments enhanced moisture diffusivity and improved polyphenol retention. Osmotic pretreatments such as pulsed vacuum osmotic dehydration (PVOD) can also improve textural integrity by reinforcing cell walls, although some anthocyanin loss may occur due to leaching. Product geometry exerts a parallel influence. Uniform slicing or controlled perforation of berry skins facilitates even heat distribution and prevents internal pressure buildup (Z.‐L. Liu et al. 2020; Nowacka et al. 2019). Ropelewska et al. (2023) successfully applied simultaneous OD and VMD to frozen, pitted whole sour cherries with uniform geometry, achieving a water activity of approximately 0.4 in 1 h. The dried products exhibited very high sensory quality, with overall quality scores above 8.8 on a 10‐point unstructured scale, as evaluated by a trained panel using quantitative descriptive analysis (QDA) (Ropelewska et al. 2023). The inclusion of suitable pretreatments and geometric optimization, therefore, contributes not only to faster water removal but also to enhanced pigment stability and overall sensory appeal.

#### 3.1.5 Energy Efficiency and Process Control

Energy efficiency represents a key technological and sustainability indicator in VMD. The VMD process can consume considerably less energy than conventional drying techniques when appropriate control strategies are employed. Automated regulation of microwave power based on real‐time temperature and pressure feedback significantly improves energy utilization by preventing overheating and ensuring uniform heat distribution throughout the product. Bórquez et al. (2015) reported that automated control of microwave power during VMD of strawberries at 700 W, reduced to 119 W after 5 min, achieved an energy efficiency of 50%–54%. This value was more than 13.5 times higher than that obtained under manual control. The resulting product exhibited a total color change (Δ*E* = 5.8) and textural quality comparable to freeze‐dried samples, and lower than that observed for conventional or manually controlled vacuum drying methods (Bórquez et al. 2015). Motavali et al. (2013) similarly reported low SEC under optimized power–pressure combinations in sour cherries (Motavali et al. 2013). Recent process‐modeling study, such as that of Cong et al. (2023), also supports the importance of multivariable optimization, showing that drying behavior and final product characteristics depend not only on microwave input but also on chamber conditions and sample positioning (Cong et al. 2023). Such findings highlight the importance of sensor‐based monitoring and adaptive power modulation in modern VMD systems, which can achieve both high process stability and superior product quality.

### 3.2 Effects of VMD on Drying Kinetics

Understanding drying kinetics is essential for interpreting the moisture migration within the fruit matrix, for optimizing process control, and for predicting product quality in VMD. The drying kinetics by VMD are governed by the combined effects of microwave‐induced volumetric heating and vacuum‐driven vapor diffusion. Unlike conventional CD or FD, where heat transfer occurs primarily from the surface inward, VMD promotes internal vaporization of water under reduced pressure, facilitating rapid diffusion of vapor through cellular pores and efficient removal from the product surface (González‐Cavieres et al. 2021; Hu et al. 2025). As a result, moisture transfer proceeds more rapidly and uniformly, while the moderate temperature under vacuum minimizes degradation of heat‐sensitive components in foods. Several studies have shown that drying behavior during VMD is best described by exponential moisture loss curves, indicating that internal diffusion is the rate‐limiting step after an initial period of constant‐rate drying. The rapid volumetric heating provided by microwaves creates a steep internal vapor pressure gradient, which significantly accelerates water migration. These mechanisms explain the drastic reduction in drying time and the superior energy efficiency typically observed in VMD compared with other drying methods. Table 2 summarizes the kinetic findings and modeling approaches reported in representative studies, highlighting the interactive effects of microwave power, operating pressure, and process configuration on drying behavior, diffusivity, and energy utilization.

**TABLE 2:** Kinetic modeling and drying behavior of vacuum microwave drying (VMD) on berries.

Reference	Material	Model/method	Key results	Remarks
Motavali et al. 2013	Sour cherry	Midilli model	*R* ^2^ > 0.99; SEC 12.9 kWh/kg; fastest drying at 1200 W and 20 kPa	Confirmed exponential moisture ratio decline; strong model accuracy
Zielinska and Michalska 2016	Blueberry	Page and Midilli	*D*_eff_ = 3.11 × 10^−^^9^ m^2^/s; hybrid HACD + VMD reduced drying time by 55%	Enhanced mass transfer via hot‐air pre‐drying
Zielinska et al. 2018	Cranberry (*Vaccinium macrocarpon*)	Weibull model	Drying followed two‐stage kinetics (constant‐rate followed by falling‐rate period); improved phenolic retention	Model accurately described microwave–vacuum drying kinetics
Zielinska and Zielinska 2019	Cranberry	Midilli	*R* ^2^ = 0.998; drying time shortened from 91 to 10 min	Optimal performance at 300 W and 5 kPa
Nowacka et al. 2019	Cranberry	RSM, page	*D*_eff_ = 2.04–2.82 × 10^−^^9^ m^2^/s	Ultrasound and PEF pretreatments improved diffusivity and phenolic retention
Z.‐L. Liu et al. 2020	Cranberry	RSM–ANN hybrid	Optimum at 150 W, 5 kPa; polyphenols 27.5 mg GAE/g DM; high FRAP activity	Intelligent modeling improved prediction accuracy
Zhou et al. 2021	Cranberry	HACD + VMD	Drying rate 17× higher than hot air; total time reduced by 54%	Hybrid process enhanced both kinetics and bioactive retention
Bórquez et al. 2015	Strawberry	Energy balance	SEC 1.7–2.3 Wh/g; 50%–54% energy efficiency	Automatic control improved sustainability and uniformity
Cong et al. 2023	Mulberry (*Morus alba*)	Two‐term, Page, Weibull models	Effective diffusivity 4.98 × 10^−8^ to 3.81 × 10^−7^ m^2^/s; optimal drying at 5.45 W/g microwave power	Two‐term model best predicted moisture loss

Abbreviations: ANN, artificial neural network; *D*_eff_, effective moisture diffusivity; DM, dry matter; FRAP, ferric reducing antioxidant power; GAE, gallic acid equivalents; HACD, hot‐air convective drying; PEF, pulsed electric field; RSM, response surface methodology; SEC, specific energy consumption; US, ultrasound; VMD, vacuum microwave drying.

#### 3.2.1 Moisture Removal and Drying Rate

The characteristic drying curve of berries subjected to VMD usually exhibits an initial short constant‐rate period followed by a prolonged falling‐rate period. During the constant‐rate stage, surface water evaporates freely under vacuum, whereas the falling‐rate stage is dominated by internal diffusion as moisture moves from the core toward the surface through capillary and vapor pathways. The moisture ratio (MR) decreases exponentially over time, reflecting the progressive resistance to moisture transfer as drying proceeds. Microwave power is the most influential factor determining the rate of moisture removal. An increase in microwave power intensity enhances the rate of vapor generation within the product, thereby accelerating internal mass transfer. Zielinska et al. (2019) demonstrated that increasing microwave power from 100 to 500 W at 5 kPa reduced the drying time of cranberries from 91 min to approximately 8–10 min, with optimal quality retention observed around 300 W (Zielinska and Zielinska 2019). Similarly, Zielinska et al. (2016) found that combining hot‐air pre‐drying at 90°C with subsequent VMD increased the effective moisture diffusivity of blueberries to 3.11 × 10^−^^9^ m^2^/s, nearly 20 times greater than that of conventional hot‐air drying (Zielinska and Michalska 2016). Motavali et al. (2013) reported comparable improvements in sour cherries and noted that the Midilli model provided the most accurate fit for experimental data, capturing both the exponential and nonlinear phases of moisture loss (Motavali et al. 2013). However, excessive microwave power can lead to local overheating or uneven drying, resulting in structural collapse or surface hardening. Therefore, a balance between power input and vacuum level is critical for maintaining product integrity and avoiding localized temperature spikes. Moderate power levels, typically between 200 and 500 W depending on material type, have been reported to provide an effective balance between drying rate and structural preservation, resulting in uniform moisture removal and reduced textural damage. These findings emphasize that controlled energy input plays a critical role in influencing drying efficiency and uniformity in VMD systems, rather than the application of maximum microwave power.

#### 3.2.2 Mathematical Modeling of Drying Kinetics

Mathematical modeling provides a theoretical foundation for describing the dynamic behavior of moisture removal during VMD. Numerous thin‐layer drying models have been employed to represent experimental data, including the Page, Henderson–Pabis, Logarithmic, Wang–Singh, and Midilli–Kucuk equations. Among these, the Midilli–Kucuk model has been recognized as the most accurate and versatile, as it successfully captures both the linear and nonlinear regions of the drying curve. Its hybrid exponential–linear form enables a precise description of simultaneous internal diffusion and surface evaporation under microwave–vacuum conditions.

Motavali et al. (2013) reported that the Midilli–Kucuk model was selected as the most appropriate one for sour cherry VMD drying kinetics, yielding correlation coefficients (*R* ^2^) greater than 0.99 and the lowest root mean square error (RMSE) values compared with other thin‐layer drying models, including Newton, Page, Henderson–Pabis, logarithmic, two‐term, and diffusion‐based models (Motavali et al. 2013). Similar accuracy was reported by Zielinska et al. (2019) and Nowacka et al. (2019) for cranberries and blueberries, with *R* ^2^ values ranging from 0.998 to 0.999. Such robust statistical fits confirm that the Midilli model effectively represents the complex drying kinetics of different berry species under varying power and pressure conditions. These models are also valuable for estimating effective moisture diffusivity (*D*_eff_) and activation energy, both of which are critical for scale‐up and process simulation in industrial VMD systems (Nowacka et al. 2019; Zielinska and Zielinska 2019). In recent years, hybrid modeling frameworks that combine response surface methodology (RSM) with artificial neural networks (ANNs) have been developed to improve predictive power and multi‐objective optimization. These models allow the simultaneous evaluation of drying rate, SEC, and quality parameters such as color and antioxidant retention. Z.‐L. Liu et al. (2020) employed a multivariable RSM–ANN approach to optimize a hybrid hot‐air convective and microwave–vacuum drying (HACD + VMD) process for cranberries. The model identified 150 W and 5 kPa as the optimal conditions for achieving maximum antioxidant retention while minimizing energy input, demonstrating the potential of intelligent modeling tools for efficient process control and parameter prediction (Z.‐L. Liu et al. 2020).

#### 3.2.3 Effective Moisture Diffusivity (*D*_eff_)

Effective moisture diffusivity (*D*_eff_) is a key kinetic parameter that quantifies the rate of internal mass transfer during drying (Man et al. 2024). In VMD systems, *D*_eff_ values typically range between 1.7 × 10^−9^ and 3.1 × 10^−9^ m^2^/s, depending on the applied microwave power, pressure level, product geometry, and pretreatment method. Higher microwave power and moderate vacuum levels generally increase *D*_eff_ by enhancing internal vapor pressure and accelerating moisture migration. However, excessive vacuum can restrict vapor escape and reduce effective diffusivity. Zielinska et al. (2016) demonstrated that combining hot‐air pre‐drying at 90°C with VMD of blueberries resulted in the highest *D*_eff_ value (3.11 × 10^−9^ m^2^/s), while Nowacka et al. (2019) reported diffusivity values between 2.0 and 2.8 × 10^−9^ m^2^/s for osmotic‐dehydrated cranberries treated with US and PEF (Nowacka et al. 2019). These results confirm that structural modification prior to drying facilitates vapor transport by creating microchannels and disrupting cellular integrity. The synergy between microwave heating and vacuum conditions leads to the generation of internal vapor pressure that effectively pushes moisture outward, thereby reducing diffusion resistance by decreasing the effective diffusion path length and tortuosity within the cellular matrix. Consequently, the drying rates in VMD are typically 10–20 times faster than those observed in conventional convective systems, while still maintaining superior retention of bioactive compounds and physical quality attributes. This combination of high diffusivity and reduced thermal load under vacuum conditions underlies the exceptional drying efficiency and quality preservation associated with VMD technology.

#### 3.2.4 Energy Efficiency and Drying Performance

VMD is distinguished not only by its fast drying rates but also by its superior energy efficiency. The volumetric nature of microwave heating ensures that energy is delivered directly to the product, minimizing heat losses to the environment. When coupled with automated process control, this approach allows for stable temperature and substantial energy savings. Bórquez et al. (2015) demonstrated that implementing automated power control during VMD of strawberry reduced SEC to as low as 1.7–2.3 Wh/g, representing a 13‐fold improvement in efficiency compared with manual operation (Bórquez et al. 2015). In a separate study, Zhou et al. (2021) found that a two‐stage HACD + VMD process reduced overall drying time by 54% compared with hot‐air drying, while the VMD stage lasted only 7–20 min. The hybrid system achieved a drying rate up to 17 times higher than conventional methods, demonstrating the synergistic advantages of combining convective and microwave–vacuum stages. On average, VMD provides energy savings of 40%–60% relative to FD and 20%–30% compared with hot‐air drying (Zhou et al. 2021). These reductions are attributed to the lower latent heat requirement under vacuum, efficient volumetric heating, and reduced process time (Calín‐Sánchez et al. 2020). Therefore, VMD emerges as a sustainable and scalable alternative for high‐quality berry dehydration, offering an optimal balance between drying rate, product quality, and energy utilization.

### 3.3 Effect of VMD on Functional Properties and Nutritional Quality of Berries

The functional and nutritional quality of dried berries is primarily determined by the retention of bioactive compounds, color, aroma, texture, and rehydration capacity. These attributes directly influence the consumer perception, health benefits, and market value of dried products. Since berries are rich in thermolabile phytochemicals such as anthocyanins, flavonoids, phenolic compounds, and vitamin C, the preservation of these components during drying is critical. Traditional drying methods often result in substantial degradation due to long exposure to high temperatures and oxygen. In contrast, VMD offers unique advantages by combining rapid volumetric heating with a low‐oxygen, low‐pressure environment that minimizes oxidation and thermal degradation. The vacuum and microwave energy create a drying environment that not only preserves nutritional compounds but also enhances structural and functional characteristics relevant to product stability and rehydration.

#### 3.3.1 Retention of Phenolic Compounds and Anthocyanins

Polyphenols and anthocyanins are among the most important compounds defining the nutritional and commercial value of berries. VMD has been proven to preserve these compounds more effectively than CD techniques by limiting oxidative degradation and shortening drying time. A comparative overview of major studies is presented in Table 3, indicating the effect of VMD on anthocyanin and polyphenol retention across different berry types.

**TABLE 3:** Summary of VMD studies on phenolic and anthocyanin retention in berries.

Reference	Material	Pressure (kPa)	Power (W)	Key results
Motavali et al. 2013	Sour cherry	20–80	360–1200	Drying 15–79 min; lowest SEC 12.9 kWh/kg at 1200 W, 20 kPa
Wojdyło et al. 2014	Sour cherry	4–6	480→120	Drying 23–48 min; 70%–90% anthocyanin retention; phenolics 7587 mg/kg dm.
Nowicka et al. 2015	Sour cherry (OD + CD + VMD)	4–6	360	High bioactive retention with 50% shorter time vs. CD
Ropelewska et al. 2023	Sour cherry (three cultivars)	3	Stepwise (100–300–250)	*a*_w_ ≈ 0.4; drying ∼1 h; sensory 8.9/10; color well retained
Zielinska and Zielinska 2019	Cranberry	5	100–500 (stepwise)	Drying 91→10 min; best retention (TP, TF, TMA) at 300 W
Zielinska and Michalska 2016	Blueberry	5	1.3 W/g	Drying 1 h; anthocyanins 30%; highest diffusivity at HACD 90°C + VMD
Zielinska et al. 2018	Cranberry (*Vaccinium macrocarpon*)	5 ± 1	100–800	High retention of phenolics and flavonoids compared with convective drying
Nowacka et al. 2019	Cranberry (US, PEF + US, OD)	6.5	150	Drying 25–38 min; best phenolics, flavonoids, vitamin C with US/PEF
Lachowicz et al. 2019	Saskatoon berry	4–6	480→120	Drying 70–80 min; anthocyanins ≈ FD; procyanidins 95% FD level

Abbreviations: *a*_w_, water activity; CD, convective drying; DM, dry matter; FD, freeze drying; HACD, hot‐air convective drying; OD, osmotic dehydration; PEF, pulsed electric field; SEC, specific energy consumption; TF, total flavonoids; TMA, total monomeric anthocyanin; TP, total phenolics; US, ultrasound; VMD, vacuum microwave drying.

Motavali et al. (2013) reported that increasing microwave power from 360 to 1200 W under 200–800 mbar reduced drying time from 79 to 15 min, with the Midilli model providing the best kinetic fit for sour cherries. Importantly, the lowest SEC (12.9 kWh/kg) was achieved at 1200 W and 200 mbar, indicating efficient mass transfer with limited quality loss (Motavali et al. 2013). Later, Wojdyło et al. (2014) observed that sour cherries dried at 480 W under 4–6 kPa retained the highest phenolic content (7587 mg/kg dm), with antioxidant activities (ABTS = 684 µmol TE/g dm; FRAP = 190 µmol TE/g dm) comparable to freeze‐dried samples, while reducing drying time to 23–48 min versus 14–40 h in CD. Hybrid approaches, combining CD and VMD, further enhanced these benefits (Wojdyło et al. 2014). Nowicka et al. (2015) demonstrated that combining OD (120 min) and convective pre‐drying (90 min) with a VMD finishing stage (4–6 kPa, 360 W) allowed sour cherries to maintain high levels of polyphenols and color stability while reducing total process time (Nowicka et al. 2015). Ropelewska et al. (2023) reported that simultaneous OD and VMD of three sour cherry cultivars at 30 ± 2 kPa with a stepwise power strategy (100–300–250–0 W per 900 s) yielded a water activity near 0.4 in 1 h, achieving excellent sensory scores (8.8–8.9/10) and color preservation (Ropelewska et al. 2023).

For cranberries, Zielinska et al. (2019) applied a stepwise power increase (100–500 W) at 5 ± 1 kPa and found that drying time decreased drastically from 91 min at 100 W to 8–10 min at 450–500 W. The optimal power (300 W) ensured the best retention of total phenolics (TP), total flavonoids (TF), total monomeric anthocyanins (TMA), and antioxidant activity (FRAP), yielding results equal to or better than FD (Zielinska and Zielinska 2019). Similarly, Nowacka et al. (2019) observed that VMD at 6.5 kPa and 150 W (25–38 min) retained phenolics, flavonoids, anthocyanins, and vitamin C significantly better than CD, particularly when US or PEF pretreatments were used (Nowacka et al. 2019). In blueberries, Zielinska et al. (2016) demonstrated that coupling HACD with VMD drastically improved process efficiency. HACD + VMD reduced drying time by 42%–55% relative to HACD alone, achieving effective diffusivity of 3.11 × 10^−^^9^ m^2^/s. The best anthocyanin retention (∼30%) and antioxidant capacity were achieved with pre‐drying at 90°C followed by VMD, showing that a brief convective stage can limit juice leakage and pigment exudation during microwave exposure (Zielinska and Michalska 2016). These studies show that moderate microwave power (2–4 W/g) and vacuum levels below 6 kPa provide the best compromise between drying kinetics and anthocyanin stability, yielding VMD products comparable or superior to freeze‐dried berries in bioactive retention.

#### 3.3.2 Vitamin C and Other Thermolabile Nutrients

Vitamin C (ascorbic acid) is highly sensitive to heat and oxygen, often serving as an indicator of overall nutritional preservation. VMD significantly improves vitamin C retention by lowering the drying temperature and limiting oxygen exposure, while maintaining short drying times. Nowacka et al. (2019) observed that cranberries dried by VMD retained markedly higher vitamin C content than convectively dried counterparts, with drying time shortened from 13 h to less than 40 min (Nowacka et al. 2019). Similarly, Z.‐L. Liu et al. (2020) reported that in a two‐stage HACD + VMD process for cranberries, polyphenols reached 27.5 mg GAE/g DM and flavonoids 3.79 mg CAE/g DM, while anthocyanin and antioxidant levels varied depending on pretreatment (ultrasonic, cryogenic, or osmotic) (Z.‐L. Liu et al. 2020). For strawberries, De Bruijn et al. (2016) and Bórquez et al. (2015) demonstrated that VMD achieved balanced moisture removal and high color stability (Δ*E* < 6), retaining approximately half of the total polyphenols and a significant portion of ascorbic acid. Despite partial anthocyanin degradation, the samples exhibited excellent flavor, acceptable texture, and shelf life over 2 months (Bórquez et al. 2015; de Bruijn et al. 2016). These findings are summarized in Table 4, emphasizing the retention of vitamin C and related antioxidants in various berry species. Therefore, VMD maintains up to 70%–85% of vitamin C and 80%–95% of total antioxidant activity, with performance comparable to FD but achieved in a fraction of the time and energy cost.

**TABLE 4:** Effect of VMD on vitamin C and overall antioxidant activity of berries.

Reference	Material	Pressure (kPa)	Power (W)	Vitamin/antioxidant results
Nowacka et al. 2019	Cranberry	6.5	150	High vitamin C and phenolic retention; 20× faster than convective drying
Z.‐L. Liu et al. 2020	Cranberry	5	150	Polyphenols up to 27.5 mg GAE/g DM; FRAP 33–47 mg TE/g DM
de Bruijn et al. 2016	Strawberry	6	700	53% polyphenols/flavonoids retained; shelf life ∼68 days
Bórquez et al. 2015	Strawberry	6–21	700→119	Δ*E* = 5.8; SEC 1.7–2.3 Wh/g; efficiency 13× higher than manual
Zhou et al. 2021	Cranberry	5	150	Polyphenols 22–28 mg GAE/g DM; antioxidant 33–48 mg TE/g DM
Zielinska et al. 2018	Cranberry (*Vaccinium macrocarpon*)	5 ± 1	100–800	High antioxidant activity associated with phenolic and flavonoid retention
Zielinska et al. 2016	Blueberry (*Vaccinium corymbosum*)	5 ± 1	1.3 W/g	Optimal antioxidant capacity obtained with HACD at 90°C, followed by VMD finishing

Abbreviations: DM, dry matter; FRAP, ferric reducing antioxidant power; GAE, gallic acid equivalents; SEC, specific energy consumption; TE, Trolox equivalents; VMD, vacuum microwave drying; Δ*E*, total color difference.

#### 3.3.3 Functional, Structural, and Sensory Quality

The functional and sensory attributes of dried berries, including color, texture, rehydration ability, and overall appearance, are key indicators of consumer acceptance and product value. VMD influences these attributes by simultaneously controlling internal vapor generation and pressure‐induced moisture removal. The combination of rapid volumetric heating and low operating pressure produces structural transformations distinct from those observed in conventional drying. As a result, VMD‐treated berries exhibit porous microstructures, reduced shrinkage, and vibrant natural color that closely resemble the characteristics of freeze‐dried fruits. A comparative summary of these quality indicators across various studies is presented in Table 5.

**TABLE 5:** Functional and sensory attributes of berries dried by VMD.

Reference	Material	Process conditions	Color/structure	Rehydration and texture	Sensory quality
Ropelewska et al. 2023	Sour cherry (*Prunus cerasus*)—three cultivars	Simultaneous OD + VMD; 30 ± 2 hPa; stepwise 100–300–250–0 W (900‐s cycles)	Vivid red color; uniform porous structure	*a*_w_ ≈ 0.4; high porosity facilitating rehydration	Excellent visual and flavor quality (8.8–8.9/10)
Lachowicz et al. 2019	Saskatoon berry (*Amelanchier alnifolia* cv. Martin, Smoky, Honeywood)	4–6 kPa; 480 → 120 W (VMD vs. FD/CD)	Improved redness (a*) and chroma; brightness similar to FD	Firm but elastic texture; no case hardening	Comparable to freeze‐dried samples
Bórquez et al. 2015	Strawberry (*Fragaria ananassa* cv. San Andreas)	47–162 mm Hg (6–21 kPa); 700 W reduced to 119 W (automatic control)	Δ*E* ≈ 5.8; minimal color loss; smooth surface	Rehydration coefficient ≈ 55%; good elasticity	Pleasant aroma and appearance maintained
de Bruijn et al. 2016	Strawberry (*F. ananassa* cv. San Andreas)	6 kPa; 700 W; 3 h at 50°C + 1 h vacuum hold	Color well preserved (Δ*E* ≈ 5); dense red tone	Rehydration volume ≈ 50% of original; acceptable firmness	Sensory score 3.5–4.3/5; good flavor retention
Zhou et al. 2021	Cranberry (*Vaccinium macrocarpon*)	HACD 80°C + VMD 150 W; 5 kPa; 7–20 min VMD stage	Glossy surface; high brightness (L*)	Stable internal structure; low shrinkage	Not reported
Zielinska and Michalska 2016	Blueberry (*V. corymbosum* cv. Bluecrop)	HACD 60°C–90°C + VMD 1.3 W/g; 5 ± 1 kPa	Enhanced blue‐purple hue; no pigment leakage	High integrity; porous and resilient matrix	Not evaluated

Abbreviations: *a*_w_, water activity; FD, freeze drying; HACD, hot‐air convective drying; OD, osmotic dehydration; VMD, vacuum microwave drying; Δ*E*, total color difference.

The microstructural features produced by VMD are closely linked to the internal vapor expansion dynamics. Rapid moisture evaporation under vacuum conditions prevents cell wall collapse and promotes the formation of microchannels that improve moisture transfer. Ropelewska et al. (2023) showed that simultaneous OD and VMD of sour cherries resulted in fruits with vivid red color, homogeneous porosity, and high sensory scores averaging 8.8–8.9 out of 10. The treated fruits retained a glossy surface and uniform structure comparable to freeze‐dried samples (Ropelewska et al. 2023). Lachowicz et al. (2019) reported that Saskatoon berries subjected to VMD exhibited increased redness (a*) and chroma values, producing an appearance nearly identical to that of freeze‐dried products (Lachowicz et al. 2019). These results indicate that the controlled internal vaporization achieved by VMD effectively preserves pigment integrity and surface smoothness, which are essential to visual quality.

**FIGURE 3:**
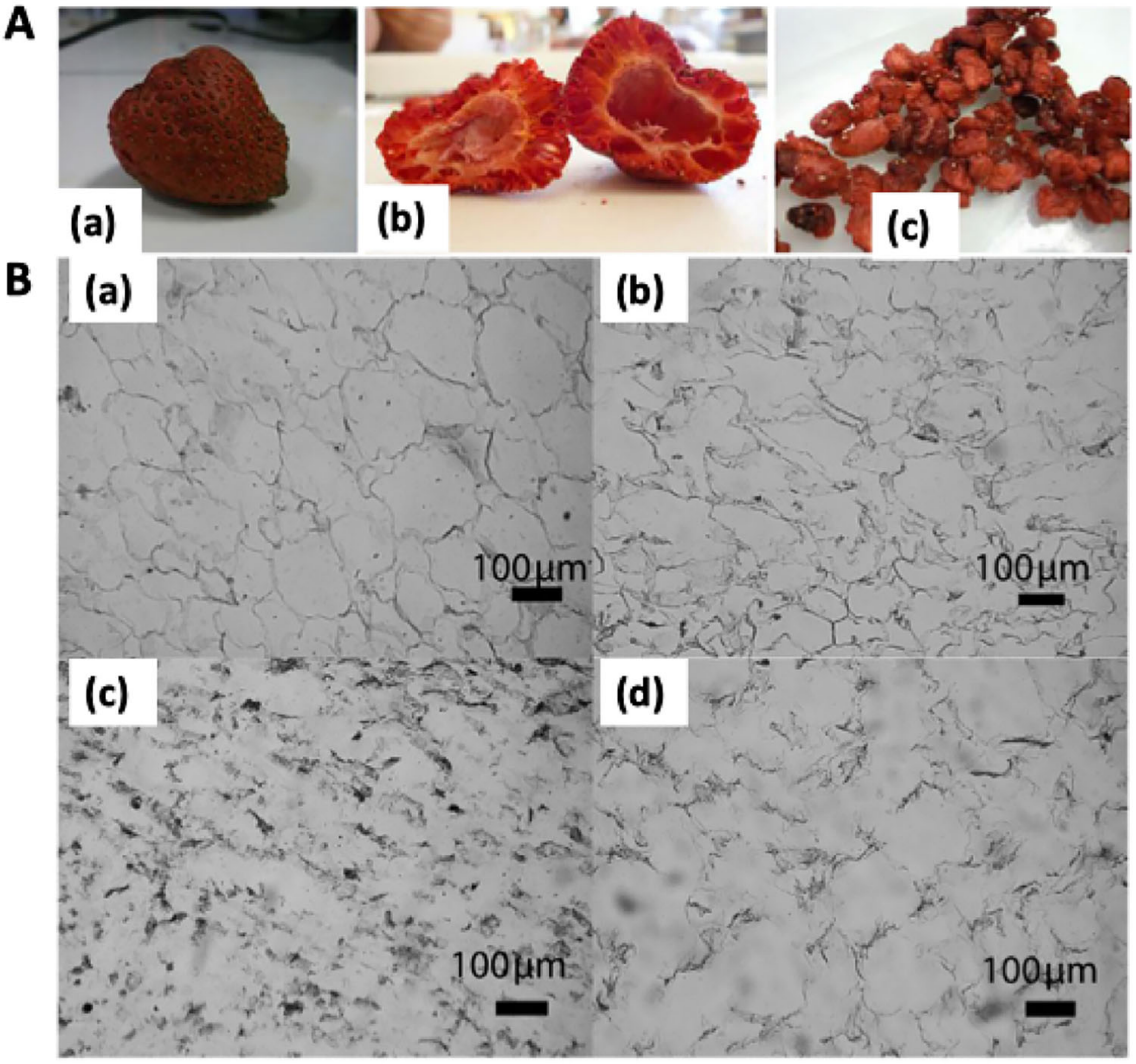
(A) Vacuum microwave drying (VMD) of strawberries under automatic temperature control (absolute pressure: 47 mm Hg; set‐point temperature: 50°C; dead band: ±1°C; microwave power: 700 W): (a) whole strawberries; (b) halved strawberries; and (c) diced strawberries. (B) Micrographs of strawberry parenchymatous tissue subjected to different drying treatments: (a) raw material; (b) vacuum microwave drying; (c) hot‐air drying; and (d) vacuum microwave drying with osmotic pretreatment. Reproduced with permission from Bórquez et al. (2015) and de Bruijn and Bórquez (2014).

The textural and rehydration characteristics of VMD‐dried berries are also superior to those obtained by traditional drying methods. The porous structure generated during microwave exposure enhances the ability of dried fruits to absorb water and recover their original softness during rehydration. Bórquez et al. (2015) found that strawberries dried under automated temperature regulation achieved a rehydration coefficient of approximately 55% and an overall color difference (Δ*E*) of about 5.8, suggesting well‐preserved tissue elasticity and minimal discoloration. These results are consistent with the macroscopic appearance shown in Figure 3A, which exhibits reduced shrinkage and relatively uniform color (Bórquez et al. 2015). De Bruijn et al. (2016) reported that rehydrated VMD‐treated strawberries recovered nearly half of their original volume after rehydration and maintained satisfactory firmness and flavor, with sensory scores ranging from 3.5 to 4.3 out of 5. As shown in Figure 3B, microscopic observations further confirm that the porous structure generated during VMD promotes water penetration while maintaining sufficient mechanical integrity, thereby contributing to favorable textural perception after rehydration (de Bruijn et al. 2014). These studies demonstrate that VMD can maintain the internal structure and water‐binding capacity of berries, resulting in products that are visually appealing and texturally pleasant.

The studies summarized in Table 5 confirm that VMD produces berries of high functional and sensory quality. VMD consistently yields products with bright, natural color, smooth surface morphology, and desirable rehydration properties. The gentle drying conditions under vacuum reduce shrinkage and maintain microstructural integrity, giving the final product an attractive appearance and a satisfying texture. Thus, the integration of microwave heating with vacuum pressure provides an efficient means of producing premium‐quality dried berries that closely resemble fresh or freeze‐dried fruits in appearance and quality.

## 4 Challenges of VMD for Berries

VMD has demonstrated considerable advantages for preserving the nutritional and sensory quality of berries; several technical challenges still limit its broader industrial implementation. These limitations arise primarily from the complex interaction between microwave radiation, vacuum conditions, and the unique structural characteristics of berry tissues.

One of the most frequently reported challenges in microwave‐based drying technologies is the heterogeneous distribution of electromagnetic energy within the drying chamber. Although microwave heating enables rapid volumetric energy transfer, the formation of standing waves and spatial variations in field intensity can create uneven heating patterns in multimode cavities (Joardder and Karim 2025; Yang et al. 2026). Such nonuniform microwave radiation has been recognized as a key limitation of microwave drying systems because it may produce localized hot spots and overheating within the product. In berry materials, this issue becomes more pronounced due to their small size, curved surfaces, and heterogeneous internal structure, which can lead to irregular microwave absorption (Zielinska et al. 2016, Zielinska et al. 2018).

Another challenge is related to the interaction between microwave radiation and the dielectric properties of berry tissues. The dielectric characteristics of fruits change dynamically during drying as moisture content decreases. This variation alters microwave penetration depth and energy absorption efficiency throughout the process (Öztürk et al. 2017; Solyom et al. 2020; Wang et al. 2026). As a result, heating behavior that is initially uniform may become unstable during later stages of drying, increasing the risk of internal temperature gradients and structural damage.

The geometry and positioning of berries inside the drying chamber also influence heating uniformity. Because berries often possess spherical shapes and thin epidermal layers, localized electromagnetic field concentration may occur at specific points on the fruit surface. Rapid internal vapor generation under vacuum can create high‐pressure gradients within the tissue, which may lead to microstructural collapse or cracking if vapor release is restricted (Cong et al. 2023; M. Li et al. 2022; H. Liu et al. 2025; Sun et al. 2026). Managing the balance between internal vapor expansion and controlled moisture diffusion, therefore, remains a critical challenge in VMD processing of whole berries.

Engineering aspects of the drying system also contribute to these limitations. The design of the resonant cavity and microwave distribution components strongly affects field stability and energy efficiency. Multimode cavities can support several electromagnetic wave modes simultaneously, producing complex and sometimes unpredictable heating patterns (Sturm et al. 2014). Various engineering strategies have been proposed to improve heating uniformity, including rotating trays, moving belts, and cylindrical rotating chambers that continuously change product orientation during drying. Product rotation has been shown to improve temperature distribution by exposing the material to microwave radiation from multiple directions (Abea et al. 2026; Yan et al. 2026).

Although rotating systems and modified cavity designs have improved process performance, many existing VMD systems have been developed through empirical adjustments rather than comprehensive mechanistic modeling. As a result, the underlying physical interactions between microwave radiation, moisture migration, and berry tissue structure remain insufficiently understood. This knowledge gap complicates the development of predictive models capable of accurately describing heat and mass transfer behavior under microwave–vacuum conditions.

Another important barrier is the scale‐up of VMD systems for industrial production. Most studies on berry drying have been conducted at laboratory or pilot scale, where product loading, microwave distribution, and vacuum control can be carefully managed. However, translating these results to industrial‐scale equipment requires optimization of power‐to‐load ratios, stable vacuum generation, and efficient vapor removal systems (González‐Cavieres et al. 2021; Nowacka et al. 2024). In addition, the complex interaction between microwave radiation and food matrices makes the design of large‐scale VMD equipment particularly challenging (Wardhani et al. 2022). Without proper system design and process control, large‐scale operations may suffer from energy inefficiency, uneven drying, or unstable operating conditions.

## 5 Future Perspectives

To overcome these challenges, future research should prioritize advanced numerical modeling approaches, including finite element and finite‐difference time‐domain simulations, to predict electromagnetic field distribution and coupled heat–mass transfer behavior in berry matrices. Improved system designs that integrate optimized cavity geometry, controlled product movement, and adaptive power modulation may enhance heating uniformity and process stability. In addition, the integration of real‐time sensing technologies, such as dielectric property monitoring and temperature feedback systems, could enable more precise control of microwave energy distribution during drying. Further investigation into the dynamic evolution of dielectric properties and physicochemical transformations occurring in berries during drying will support the development of scientifically grounded process control strategies. In particular, future studies should explore how microwave–vacuum conditions influence the microstructural integrity of berry tissues and the retention, stability, and bioaccessibility of bioactive compounds such as anthocyanins and phenolic compounds. Moreover, the application of data‐driven approaches, including machine learning and predictive modeling, may facilitate the optimization of drying parameters and improve process efficiency. Finally, structural optimization and energy‐efficient system configurations, including improved airflow management, enhanced power control algorithms, and scalable equipment design, will be essential to increase reliability, reduce operational costs, and enable the industrial implementation of VMD technology for berry processing.

## 6 Conclusion

VMD has emerged as a highly efficient dehydration technology for thermally sensitive fruits such as berries. VMD combines rapid volumetric microwave heating with reduced‐pressure evaporation, enabling efficient moisture removal at relatively low temperatures while minimizing degradation of thermolabile compounds such as anthocyanins, phenolics, and vitamin C. Compared with conventional drying techniques, VMD provides an effective balance between product quality and energy efficiency. This review summarizes recent advances in the application of VMD to berry processing, highlighting the influence of key operational parameters, drying kinetics, and process configurations on product quality. Hybrid drying strategies and appropriate pretreatments have demonstrated considerable potential to enhance drying efficiency and bioactive compound retention. Despite these advantages, several challenges remain, particularly related to microwave energy distribution, dynamic changes in dielectric properties, and the scale‐up of VMD systems for industrial production. Future research should therefore focus on improved system design, advanced numerical modeling of heat and mass transfer, and the integration of real‐time monitoring and adaptive control strategies. These developments will be essential for improving process reliability and facilitating the broader industrial implementation of VMD technology for high‐value berry products.

## Author Contributions

**Long Phuoc Lieu**: conceptualization, investigation, writing – original draft, methodology, validation, visualization, software, formal analysis, data curation, resources. **Youngsoo Lee**: writing – review and editing, funding acquisition, supervision, project administration, data curation, visualization, validation, investigation, conceptualization.

## Conflicts of Interest

The authors declare no conflicts of interest.
